# Maternal and Neonatal and Child Health Priorities in Africa and Asia

**DOI:** 10.1155/2016/9107970

**Published:** 2016-09-18

**Authors:** Sten H. Vermund, Renu Garg, Ying-Ru Lo, Emily K. Sheldon, Kasonde Mwinga

**Affiliations:** ^1^Vanderbilt Institute for Global Health and Department of Pediatrics, Vanderbilt University School of Medicine, Nashville, TN 37203, USA; ^2^WHO Regional Office for South-East Asia, New Delhi 110 002, India; ^3^WHO Regional Office for the Western Pacific, 1000 Manila, Philippines; ^4^WHO Regional Office for Africa, Brazzaville, Congo

Immense challenges remain in the field of maternal, neonatal, and child health (MNCH) in Africa and Asia. Vast rural poverty continues to mire both continents in patterns of risk for pregnant women and their children [1]. Especially in Africa, many nations failed to meet Millennium Development Goals 4 and 5 [2–5]. The aspirational Sustainable Development Goals also will be difficult to achieve. Still, much is being accomplished, as noted by the falling under-five mortality rates in all but a few countries [6–10].

For decades in its global health coordination role, the World Health Organization (WHO) has highlighted the vital challenges in ensuring the welfare of women and children. The maternal, neonatal, and child health (MNCH) arena offers both preventive opportunities and obstacles; successful programmatic models show immense promise for reducing disease and suffering in low and middle income countries (LMIC). In 2015, we solicited manuscripts from global health investigators working in low and middle income countries (LMICs) in either Africa or Asia whose work would be suitably highlighted within a special 2016 issue of this journal. This commentary introduces the topics in this special issue, reflecting a diversity of topics and methodologies in the MNCH field.

As one reads the papers in this special issue, a number of themes emerge. The imperative to enable mothers and fathers to space and limit their children's births through contraception is presented by several investigators, a topic that is dramatized by the 7.3-billion world population as of this writing (June 2016). Similarly, the urgent need for quality improvement and equitable distribution of health care workers (HCW) is presented through various prisms. The continuing challenges of maternal sexually transmitted and other infections are evident in both Asia and Africa. The continuing threat of infectious diseases in neonates and children was also highlighted in this issue.

We hope that future articles on innovative approaches to improving maternal literacy, including health literacy and numeracy, use of modern digital devices to enhance linkage and adherence to care, combination prevention approaches, or economic empowerment of women, will be presented from Africa and Asia [11–13]. MNCH is more likely to improve when integrated development programming replaces the streaming of funds into “silos” of health or education or economic development [14–17].

The world faces perils of global climate change, loss of biodiversity, overpopulation, desertification, the rise of the megacities, and water shortages. Globalized trade and marketing are degrading healthy traditional diets with fatty, fried “fast foods” and the aggressive marketing of tobacco. With progress towards the control of infectious diseases and the rise of noncommunicable diseases in LMICs, new challenges are facing mothers and children, including obesity and second-hand smoke exposure. Civil strife, territorial aggression, war, and nuclear threats continue. Global educational, economic, and health inequities persist and, in some nations, worsen. Violence against women and children, suppression of women's rights, and denial of school opportunities for girls remain plagues in both continents. Yet progress towards improved MNCH is undeniable. The guest editors of this special issue posit two questions that build upon the reports of progress highlighted here: “How can we expand successes in women's and children's health? How can we improve the world that a new, healthier generation will inherit?”

*Sten H. Vermund* *Renu Garg* *Ying-Ru Lo* *Emily K. Sheldon* *Kasonde Mwinga*
